# Correlation of MADRS and Q-LES-Q-SF in patients with Bipolar II depression: A post-hoc analysis of an Indian Phase 3 study

**DOI:** 10.1192/j.eurpsy.2025.1071

**Published:** 2025-08-26

**Authors:** A. Dharmadhikari, P. K. Chaurasia, Y. Patel, D. Choudhary, P. L. Dasud, M. Bhirud, P. S. Meena, F. Shah, G. Ganesan, B. P. S. Rathour, K. Mistry, M. Dutta, A. Ramaraju, S. G. Goyal, S. B. Mangalwedhe, G. Kulkarni, A. Mukhopadhyay, P. Chaudhary, G. T. Harsha, M. Parikh, S. Dey, S. Sarkhel, N. U. Jyothi, A. Kumar, N. K. Sooch, S. Saha, A. Shetty, P. H. Devkare, A. Shetty, D. Patil, P. Ghadge, A. Mane, S. Mehta

**Affiliations:** 1 Shree Ashirwad Hospital, Dombivali; 2 Gangoshri Hospital, Varanasi; 3 VS General Hospital, Ahmedabad; 4 GSVM Medical College, Kanpur; 5 Global 5 Hospital, Vashi; 6 Dhadiwal Hospital, Nashik; 7 Jawahar Lal Nehru Medical College, Ajmer; 8 Health 1 Super Speciality Hospital, Ahmedabad; 9 Medstar Speciality Hospital, Bangalore; 10 Atmaram Child Care and Critical Care Hospital, Kanpur; 11 Prajna Health Care, Ahmedabad; 12 Om Hospital, Raipur; 13 Harshamitra Super Speciality Cancer Center and research institute, Trichy; 14 S. P. Medical College & A.G. Of Hospitals, Bikaner; 15 Karnataka Institute of Medical Sciences, Hubli; 16 Manodnya Nursing Home, Sangli; 17 Nil Ratan Sircar Medical College and Hospital, Kolkata; 18 GMERS Medical College, Ahmedabad; 19 Rajlaxmi Hospital, Bangalore; 20 B.J. Medical College and Civil Hospital, Ahmedabad; 21 Sparsh Hospital, Bhubaneswar; 22 IPGME&R and SSKM Hospital, Kolkata; 23 Government General Hospital, Guntur; 24 S N Medical College, Agra; 25 Dayanand Medical College & Hospital, Ludhiana; 26 Sun Pharma, Mumbai, India

## Abstract

**Introduction:**

Lumateperone, an atypical antipsychotic drug approved in 2021 for bipolar depression, has a dual mechanism of action by combination of activity at central serotonin (5-HT2A) and dopamine (D2) receptors. In India, Quetiapine is one of the approved drugs for use in depressive episodes for bipolar disorder.

**Objectives:**

This post-hoc analysis of an Indian Phase 3 study was conducted to evaluate the correlation of severity of depression assessed via Montgomery-Asberg depression rating scale (MADRS) and quality of life assessed via Quality-of-life enjoyment and satisfaction-short form questionnaire (Q-LES-Q-SF) when treated with Lumateperone 42mg or Quetiapine 300mg.

**Methods:**

The phase-III, randomized, multi-centric, assessor-blind, parallel-group, active-controlled, comparative, non-inferiority study included patients with Bipolar II depression with moderate severity having a MADRS score ≥20 and Clinical global impression–bipolar version–severity (CGI-BP-S) score ≥4. The study was conducted after receiving regulatory and ethics committee approvals. The patients were randomized (1:1) to either receive Lumateperone 42mg [Test] or Quetiapine 300mg [Comparator] for 6 weeks. In this post-hoc analysis, correlation between MADRS and Q-LES-Q-SF were evaluated and for safety outcomes treatment emergent adverse events (TEAEs) were assessed. [Clinical trial registration: CTRI/2023/10/058583]

**Results:**

This post-hoc analysis included 462 patients [231 each in Test and Comparator]. The baseline demographic characteristics were comparable in between treatment arms. The Pearson’s correlation coefficient between change from baseline in MADRS score and Q-LES-Q-SF score was statistically significant for both treatment arms at Day 42 [Test: -0.192, p=0.0043; Comparator: -0.299, p<0.0001] and the linear regression between 2 arms was not statistically significant (p=0.0853), indicating weak negative correlation between the 2 scales [Figure 1 and Figure 2]. The incidence of TEAEs were similar in both treatment arms [Test: 34.6%; Comparator: 35.5%] and no serious adverse events were reported.

**Image 1:**

**Figure:**
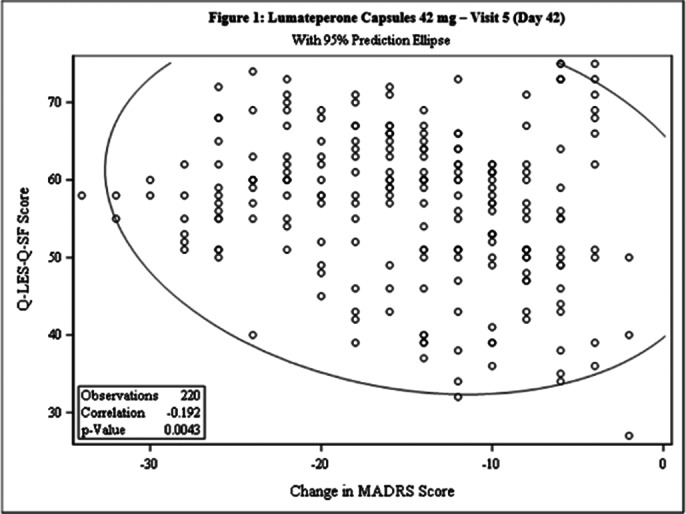

**Image 2:**

**Figure:**
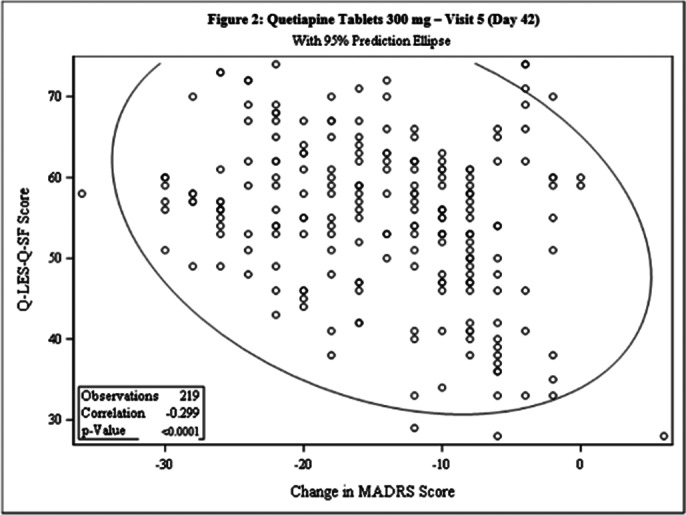

**Conclusions:**

This post-hoc analysis demonstrated that patient with Bipolar II depression when treated with Lumateperone 42mg or Quetiapine 300mg, the reduction in MADRS score is inversely proportional to Q-LES-Q-SF score.

**Disclosure of Interest:**

A. Dharmadhikari: None Declared, P. Chaurasia: None Declared, Y. Patel: None Declared, D. Choudhary: None Declared, P. Dasud: None Declared, M. Bhirud: None Declared, P. Meena: None Declared, F. Shah: None Declared, G. Ganesan: None Declared, B. P. Rathour: None Declared, K. Mistry: None Declared, M. Dutta: None Declared, A. Ramaraju: None Declared, S. G. Goyal: None Declared, S. Mangalwedhe: None Declared, G. Kulkarni: None Declared, A. Mukhopadhyay: None Declared, P. Chaudhary: None Declared, G. T. Harsha: None Declared, M. Parikh: None Declared, S. Dey: None Declared, S. Sarkhel: None Declared, N. Jyothi: None Declared, A. Kumar: None Declared, N. Sooch: None Declared, S. Saha Employee of: Sun Pharma, A. Shetty Employee of: Sun Pharma, P. Devkare Employee of: Sun Pharma, A. Shetty Employee of: Sun Pharma, D. Patil Employee of: Sun Pharma, P. Ghadge Employee of: Sun Pharma, A. Mane Employee of: Sun Pharma, S. Mehta Employee of: Sun Pharma

